# Creating accessible and inclusive undergraduate studentship opportunities: the ENRRICH experience

**DOI:** 10.1186/s12909-025-06847-y

**Published:** 2025-02-16

**Authors:** Robert Beattie, Kelly Russell, Kristy Wittmeier, Jenna Mitchell-Dueck, Kristene Cheung, Mojgan Rastegar, Alana Slike, Valerie Williams, Ming-Ka Chan, Mary Wilson, JLP Protudjer

**Affiliations:** 1 Department of Biochemistry and Medical Genetics, Rady Faculty of Health Sciences, University of Manitoba, Winnipeg, MB Canada; 2 Children’s Hospital Research Institute of Manitoba, 501G-715 McDermot Avenue, Winnipeg, MB R3E 3P4 Canada; 3 Department of Pediatrics and Child Health, Rady Faculty of Health Sciences, University of Manitoba, Winnipeg, MB Canada; 4 Rehabilitation Centre for Children, Winnipeg, MB Canada; 5 Department of Clinical Health Psychology, Rady Faculty of Health Sciences, Max Rady College of Medicine, University of Manitoba, Winnipeg, MB Canada; 6 Department of Pharmacology and Therapeutics, Rady Faculty of Health Sciences, University of Manitoba, Winnipeg, MB Canada; 7 Equity, Access, and Participation Office, Rady Faculty of Health Sciences, University of Manitoba, Winnipeg, MB Canada; 8 Department of Food and Human Nutritional Sciences, University of Manitoba, Winnipeg, MB Canada; 9 Institute for Environmnetal Medicine, Karolinska Institutet, Stockholm, Sweden

**Keywords:** Equity, diversity and inclusion, Representation, Studentships

## Abstract

Recognizing the systemic exclusion of structurally oppressed groups from academic awards, the ENRRICH (Excellence in Neurodevelopment and Rehabilitation Research In Child Health) summer studentship emphasized the inclusion of structurally oppressed groups. Herein, we outline the processes in creating this funding opportunity, and plans for improvement, including enhanced representation among supervisors.

## Background

Undergraduate summer studentships are important funding sources and provide unique opportunities for research exposure. Unlike loans, studentships are typically non-repayable [1]. Undergraduate studentships are largely merit-based, focusing on academics, athletics, and extracurriculars [1]. Merit-based funding opportunities often preferentially support students from privileged groups [1]. Some studentship applications require relevant community service or leadership experience. Students from lower-resourced settings may be systemically excluded due to volunteer requirements and/or potential nepotism within such activities. Exclusion from studentships results in income and opportunity losses and can perpetuate inequities, as completing a studentship can provide demonstrable academic gains (e.g., presentations, awards, publications) [2, 4]. Such accomplishments may contribute to the success of future funding or graduate school applications. Relationships developed through studentships can support future employment, and ignite and preserve students’ research interests [3, 5]. Failure to render summer studentships accessible to structurally oppressed groups^1^ may perpetuate Eurocentric curricula and biased work and learning environments [6]. This paper describes the development of an undergraduate summer studentship program rooted in equity, access, and participation (EAP) and guided by academics, students, people with lived experience, and an Indigenous Knowledge Keeper. The studentship is situated within ENRRICH, the Excellence in Neurodevelopment and Rehabilitation Research in Child Health theme. Established in 2018 within the Children’s Hospital Research Institute of Manitoba (CHRIM), ENRRICH aims to generate and translate research to improve the health of children and youth with neurodevelopment and rehabilitation needs in the Manitoba, and surrounding catchment areas. By rooting this summer studentship in EAP, we attempt to overcome the abovementioned limitations associated with merit based funding. Specifically, we aimed to develop an undergraduate summer studentship application process that removed cost and process barriers while offering cross-disciplinary research opportunities to students interested in neurodevelopment or rehabilitation and who self-identified as being from one or more systemically-excluded communities.

## Methods

In April 2022, ENRRICH initiated discussions on studentship equity, sparked by concerns about students being ineligible for most studentships due to not meeting GPA requirements. These discussions coincided with a presentation from the University of Manitoba’s Office of EAP on academia’s lack of representation. ENRRICH members were invited to collaboratively explore solutions. An eight-member studentship committee was formed, including academics, the ENRRICH coordinator, and a graduate student. The committee is ethnically diverse and disproportionately women.

The committee engaged with Manitoba-based EAP experts through a series of virtual meetings and subsequent email communication. The impactful contributions of the EAP experts are reflected in their listings as authors of this manuscript. Their feedback emphasized the use ofinclusive language (such as gender-neutral pronouns and plain language text), and inviting applicants to share lived experiences relevant to this studentship application. In contrast, they encouraged the removal of official transcripts (for which there is an associated fee) as well as lengthy letters of support from supervisors and referees. As this initiative was an educational endeavour, the University of Manitoba’s Health Research Ethics Board did not require consent to be obtained.

Ultimately, studentship criteria were created without consideration to academic standing so long as students were not on academic probation (Table 1). Transcripts were not required, removing cost and process barriers [7, 8]. Students were asked to self-declare identities within one (or more) structurally oppressed group(s). Informed by language that was being used at the time within the University of Manitoba and CHRIM, we broadly define these groups to include anyone with a minoritized identity including gender, ethnicity, and/or disability.

**Table 1:** ENRRICH summer studentship application requirements

Student requirements	Supervisor requirements
Selections will be made by a two-round lottery system. Round 1 is designated for individuals who are members of Black, Indigenous, and/or racially marginalized groups, including those with disabilities, and/or persons of sexual and/or gender diverse identities. Round 2 will include all applicants who meet the eligibility criteria.• • Be enrolled in a post-secondary educational program in Winnipeg or • Have recently graduated in the six months prior to the start of the studentship (i.e., in December or April) • Not hold another award concurrently Applicants are asked to self-identify in their cover letter aspects of their identity that position them to bring currently under-represented viewpoints, expertise, and forms of excellence. Applicants with prior degree(s) or diploma(s) will be considered if they are enrolled in another undergraduate program at the time of application.	Supervisors must be ENRRICH members (if the potential supervisor is not yet an ENRRICH member, they must apply before the studentship deadline). Supervising ENRRICH members must: • Submit a personal statement including their demonstrated commitment and approach to issues pertaining to anti-racism and social justice (including principles of equity, access, and participation) in their teaching, research, service, and/or other experiences. • This personal statement includes past contributions and plans going forward. • Describe the project the student will work on, and the mutually agreed upon goals that the proposed student would like to gain during the studentship. • Explain how they will incorporate the proposed student into their broader research group or lab. • Participate in EAP training for supervisors prior to the student beginning their summer research term.

### Student application process, supports, and expectations

With the support of our institutional communications team which provided technological support and opportunities for broader dissemination, we hosted a virtual town hall to share information about this new studentship. A maximum of four studentships were available and of same monetary value as other (merit-based) undergraduate studentships within the affiliated organizations (University of Manitoba, CHRIM).

To further support potential students, we also published a blog post titled, “*Tips on how to find and approach a potential supervisor for summer studentships*” on the ENRRICH website. Biographies of potential supervisors were available for students to review on the ENRRICH and CHRIM websites.

To further support potential supervisors, ENRRICH investigators received information about their eligibility criteria and responsibilities. rs. Notably, we developed a policy that a researcher could only have one student receive an ENRRICH studentship in a given competition.

The application process was designed to place greater responsibility on the potential supervisor and less burden on the student, as the supervisor completed most of the application. Before applying, students needed to meet their potential supervisor and discuss a summer research project. The application had open-ended questions for students to briefly describe their career goals and relevant skills obtained through life, work, volunteer, or academic activities. Students summarized what receiving this studentship would provide them that would not otherwise be accessible. Supervisors had to describe their commitment to EAP principles and outline the proposed project. By signing the application, the supervisor attested that the student qualifies for the studentship. Potential supervisors could sponsor more than one student; however, as described above, only one of their applicants could receive a studentship. To promote a culturally safe environment for the students, supervisors of awarded students were required to attend formal EAP training prior to the start of the studentship.

The application period was from January 10th to March 10th _,_ 2023. Awards were announced on March 15th _,_ 2023, and studentships began in May. This timeline was developed to provide sufficient time for advertising, applying, and for awardees and non-awardees to plan their summer.

We received a total of 18 applications,, which represented eight supervisors (three women; five men), from both clinical research (*n* = 3) and fundamental research (*n* = 5). In each of the two rounds, two students were selected. Round 1 included only applicants who self-identified as a member of structurally-oppressed groups (*n* = 15). Round 2 included all applicants; with the exception of those who were selected in the Round 1 draw. In adherence with our policy of each supervisor only supervising one student, Round 2 also excluded any students who applied to work with supervisors whose students were selected in Round 1. After applying these criteria, there were 10 eligible applicants for Round 2.

The two-round random draw selection process ultimately yielded the four successful applicants, all of which were awarded to students in the fundamental sciences. Students were welcomed into their supervisor’s lab and were either took on a small research project, or a portion of a larger project.

At the start of the summer, students were connected with the Knowledge Keeper who works with ENRRICH, and the ENRRICH Coordinator, as part of the orientation and to facilitate relationship building should students wish to seek additional support during their studentship.

In addition to their research activities, students attended an ENRRICH member-wide cultural awareness activity, joined ENRRICH’s summer membership meetings, and participated in a presentation practice session. Students also presented their work at the annual CHRIM summer student presentation series.

At the mid-point of the studentship term, the ENRRICH coordinator checked in with all students. At the end of the term, the studentship committee’s student representative or the ENRRICH coordinator conducted informal exit interviews with each of the four students.

### Reflections on studentship outcomes

Our first iteration of this summer studentship program had notable strengths. The program brought together dedicated student researchers and supervisors. Students had learning opportunities and developed skills in scientific presentation, data analysis, laboratory techniques, and adaptability in research, and cultural competency. Collectively, these are all tools necessary to begin to tackle the challenges of scientific inquiry.

Exit interviews and committee’s reflections provided insights into some areas of improvement for future iterations of summer studentship program. First, related to supervisors, we will work to ensure all ENRRICH members (not just awardee supervisors) participate in ongoing anti-oppression training. A weighted selection process will be used, so that supervisors who only apply with one student are not disadvantaged in the draw, relative to a supervisor who applies with multiple students. For example, if the highest number of students submitted by a single supervisor is four, then supervisors with only one student will receive four ‘entries’ into the draw to ensure that student’s supervisor has the same chance of being selected as the four students who selected the same supervisor. To further support representation within the supervisor group, we will encourage co-supervision, to share the work of supervising a summer student. Finally, to enhance support of students who self-identify as a member of one or more marginalized groups, we will explore mentorship opportunities with academic faculty, senior learners, or mentors from related disciplines, who identify similarly.

Second, to clarify the application and selection process, we will develop a “Frequently Asked Questions” summary for the studentship and share this information through the ENRRICH website and CHRIM communications.

Third, upon review, some applicants had previously worked with their potential supervisor. This provides an unfair advantage and mitigates the intent of the studentship to provide new opportunities for students. Henceforth, those who have previously worked with their proposed supervisor will be ineligible to apply with the same supervisor.

Fourth, there were few Indigenous student applicants despite efforts to advertise and share information with Indigenous student groups and organizations. We aim to broaden our communication network and where possible, partner with Indigenous-led organizations to increase engagement with Indigenous students.

Like many new initiatives, the first iteration of this program leaves room for growth. Pending adequately large numbers of students who receive funding from our studentship program, we intend to conduct a mixed methods longitudinal evaluation. Estimating 4 students per year, we expect to complete our evaluation process in 5–6 years, to allow for data collection and robust analysis and limit the possibility of identification of single student or supervisor.

To additionally understand the impact of the ENRRICH studentship, we will explore developing a consent-based system to follow participants’ long-term academic/professional progress. To enhance the impact and reach of the ENRRICH summer studentship program, future iterations may explore partnerships for expanding program funds. Collaborating with industry could tap into Corporate Social Responsibility initiatives and increase the number of available studentships. Scientific societies, government bodies/public health organizations, non-profit organizations, and charitable foundations could yield additional support. Alumni networks and private donors could be leveraged. Any new collaboration must align with ENRRICH’s commitment to support EAP in academia. Sustainable and long-term partnerships will support future growth and success.

## Conclusion

The ENRRICH summer studentship program, designed to enhance EAP in academic research, signifies an equity-informed approach to awarding summer studentships. Continuous evaluation and adaptations are necessary. By building on strengths and addressing areas for improvement, the program can better fulfill its mission of supporting an anti-oppressive and equitable academic environment.

## Data Availability

No datasets were generated or analysed during the current study.

## Abbreviations

2SLGBTQIA +: Two-spirit, lesbian, gay, bisexual, transgender, queer and/or questioning, intersex, asexual, and the plus reflects the countless affirmative ways in which people choose to self-identify
CHRIM: Children’s Hospital Research Institute of Manitoba
CIHR: Canadian Institutes of Health Research
EAP: Equity, access, and participation
ENRRICH: Excellence in Neurodevelopment and Rehabilitation Research in Child Health

## Footnotes

1 We used language that aligned with our organization at the time of writing, regarding principles of equity, access, and participation. We recognize these terms may change and evolve over time, and we plan to evolve the language associated with the studentship accordingly.
